# The Togo national proficiency test pilot programme for basic clinical chemistry tests

**DOI:** 10.4102/ajlm.v11i1.1565

**Published:** 2022-06-24

**Authors:** Kafui C. Kouassi, Améyo M. Dorkenoo, Komivi Gbada, Yaovi-Gameli Afanyibo, Minogblon Têko, Adjane Koura

**Affiliations:** 1 Unity of External Quality Assessement, Division of Laboratories, Ministry of Health and Public Hygiene, Lomé, Togo; 2 Medical and Biological Analysis-Biochemistry, Higher School of Biological and Food Techniques, University of Lomé, Lomé, Togo; 3 Division of Laboratories, Ministry of Health and Public Hygiene, Lomé, Togo; 4 Department of Health Sciences, Faculty of Health Sciences, University of Lomé, Lomé, Togo; 5 Lomé Commune Regional Hospital, Ministry of Health and Public Hygiene, Lomé, Togo; 6 National Institute of Hygene, Ministry of Health and Public Hygiene, Lomé, Togo; 7 Bè Secondary Hospital, Ministry of Health and Public Hygiene, Lomé, Togo; 8 Division of Laboratories – RESAOLAB, Ministry of Health and Public Hygiene, Lomé, Togo

**Keywords:** quality control, biochemistry, laboratory, performance, Togo

## Abstract

**Background:**

A national proficiency test (PT) programme is not currently implemented in most low-income countries. However, participation in such PT programmes assists improves test performance and result accuracy.

**Objective:**

This study assessed how well 11 government hospital laboratories performed 18 basic clinical chemistry tests and identified areas needing improvement.

**Methods:**

A cross-sectional study was carried out by the Division of Laboratories of the Ministry of Health of Togo from 01 July 2016 to 31 December 2016. The test performance was evaluated using panels provided by One World Accuracy, Canada (Vancouver). The Clinical Laboratory Improvement Amendments criteria were used in evaluating the laboratories, and their success rates were compared with the World Health Organization Regional Office for Africa’s target of 80%.

**Results:**

The overall rate of acceptable results at the laboratories was over 80% for glucose, alanine aminotransferase, aspartate aminotransferase, gamma-glutamyltransferase, alkaline phosphatase and triglycerides tests. The laboratories using fully automated spectrophotometers had an acceptable results rate of 89% (*p* = 0.001). The overall performance of the laboratories by cycles varied from 71% to 82%.

**Conclusion:**

This national PT programme identified the tests, which laboratories must improve their performance (urea, creatinine, uric acid, bilirubin, cholesterol, total protein, calcium, magnesium, phosphorus). It demonstrated the need for the use of routine appropriate internal quality control in all laboratories. The proficiency test programme should be extended to all clinical laboratories and target all biology disciplines.

## Introduction

Clinical laboratory test results are for screening, diagnosis, prognosis, therapeutic monitoring of chronic pathologies and epidemiology surveillance.^1^ These results are more reliable when internal quality control (IQC) and external quality assessment (EQA) are implemented in the clinical laboratories. A proficiency test (PT) is one form of EQA that uses pre-established criteria to evaluate the performance of a participating laboratory (PL) compared with other laboratories.^2^ The inter-laboratory comparison, audit, and accreditation culture has not yet taken root in low-income countries, such as Togo, with a Gross National Income per capita of $1035 or less. A 2013 survey reported that more than 90% of African countries had no accredited laboratories meeting the International Organization for Standardization standard 15189 (ISO 15189), the international quality standard for clinical laboratories.^3,4^

Clinical biochemistry tests remain predominant in the management of pathologies, whether resulting from transmissible or non-transmissible diseases.^5,6^ It is therefore essential that the results of these clinical chemistry tests are accurate. The participation of government hospital laboratories in a national PT programme as required by the ISO 15189 standard could help to ensure accurate test results.^7^ In Togo, the clinical diagnostics laboratories face many challenges, the most important of which are: obtaining market authorisation from distributors of *in vitro* diagnostic medical devices; implementing appropriate quality assurance processes, including IQC, and participating in a national or private EQA (PT) programme; and getting the ISO 15189 accreditation (particularly for the national reference laboratories). Since 2012, only three clinical laboratories in Togo have been ISO 15189:2012 accredited, two within the public health institute by the West African Accreditation System and one private laboratory by the French accreditation committee.^8,9^ After a successful PT feasibility assessment in 2012 involving 11 clinical laboratories across the Lomé municipalities, in 2016, the Division of Laboratories, Ministry of Health, Togo, implemented a national PT programme.^10^ The RESAOLAB Project (West African Network of Clinical Laboratories) implemented the PT programme, supported by Fondation Mérieux.^11^ In total, the health system of Togo is composed of 179 government hospital laboratories organised in a tiered system, depending on their capabilities. A pilot phase of this programme was initiated the same year and involved 11 government hospital laboratories representing the central, intermediate and peripheral laboratory levels. The study aimed to assess how well these 11 government hospital laboratories performed 18 basic clinical chemistry tests and to identify areas where improvement may be required.

## Methods

### Ethical considerations

This study did not involve human subjects or animal research. This PT programme is part of regular assessment activities of the Ministry of Health of Togo and does not require any particular ethical consideration. A unique PT biological material was obtained from the Canadian company One World Accuracy and sent to all participating laboratories. The PT programme is part of the Ministry of Health’s regular assessment activities and does not require a particular ethical consideration. More so, the PT activity did not violate the current ethical considerations of the Declaration of Helsinki.

### Study design

A cross-sectional assessment was carried out from 01 July 2016 to 31 December 2016. The programme comprised four cycles at the rate of one cycle per month from August to November 2016. The study was conducted in three steps: (1) identification and training of participating laboratories (PLs); (2) selection of tests, reception of samples at the central level, and dispatch of samples to PLs; (3) collection of PT results and data evaluation.

### Participating laboratories and personnel training

Eleven government hospital laboratories (representing 32% of 34 laboratories that routinely perform the 18 basic clinical chemistry tests), were enrolled in the PT programme. These 34 government hospital laboratories represent 19% of all the government hospital laboratories spread across the six health regions in Togo. The PLs were purposefully selected to ensure that all three levels of the health system were represented: three University Hospital Laboratories for the central level; six Regional Hospital Laboratories for the intermediate level; and two District Hospital Laboratories for the peripheral level. These PLs were randomly anonymised by numbering them 1–11. None of these PLs had ISO/International Electrotechnical Commission (IEC) 15189 accreditation. A unique PT panel was obtained from Oneworld Accuracy^®^ (OWA) located in Vancouver, British Columbia, Canada, and shipped by Fedex^®^ Expedition Services. Following arrival and customs clearance, the package was immediately sent to the Institut National d’Hygiène, the national public health reference laboratory in Togo, which is ISO 15189 accredited. At Institut National d’Hygiène, sample integrity and adherence to temperature requirements (2 °C – 8 °C) were confirmed using a calibrated thermometer. These samples were stored for up to 24 h at 2 °C – 8 °C at the Institut National d’Hygiène and then sent to the PLs for immediate testing upon reception. Samples were transported refrigerated in individual insulated containers to each PL. The maximum transit time was 12 hours for the furthest PL.

Two laboratory technicians from each PL were trained by two members of the coordinating team of the national PT programme of the Division of Laboratories, Ministry of Health of Togo. The training was provided by members with experience in the areas of quality management, statistical analysis, and biochemical analysis. The PL technicians were trained on PT principles under ISO/IEC 17043, IQC and the inter-laboratory comparison requirements in ISO/IEC 15189, and OWA^®^ PT PL guidelines for demonstration.^2,7,12^ Training on the use of the Oneworld Accuracy System software platform (Collaboration Secretariat of Oneworld Accuracy Group, Vancouver, British Columbia, Canada), for setting measurement units, methods, equipment and test result submission, was also provided.

### Laboratory tests and samples

The 18 basic clinical chemistry tests and serum analytes selected by the Ministry of Health Division of Laboratories for this PT programme were: urea, blood glucose, creatinine, uric acid, alanine aminotransferase (Enzyme Commission [EC] number: 2.6.1.2 [International Union of Biochemistry and Molecular Biology, https://iubmb.qmul.ac.uk]), aspartate aminotransferase (EC 2.6.1.1), gamma-glutamyl transferase (EC 2.3.2.2), alkaline phosphatase (EC 3.1.3.1), total bilirubin, direct bilirubin, total cholesterol, high-density lipoprotein cholesterol, low-density lipoprotein cholesterol, triglycerides, total protein, calcium, magnesium, and phosphorus. These tests were performed by the technicians of the 11 PLs using commercially purchased reagents and IQC was used where available. Analytical methods used for each test were recorded.

The 11 PLs used the same methods but not the same vendor kits for the following tests: urea (urease), blood glucose (glucose oxidase-hydrogen peroxide), creatinine (alkaline picrate), uric acid (uricase-hydrogen peroxide), alanine aminotransferase (International Federation of Clinical Chemistry and Laboratory Medicine method without pyridoxal-5-phosphate cofactor), aspartate aminotransferase (International Federation of Clinical Chemistry and Laboratory Medicine method without pyridoxal-5-phosphate cofactor), gamma-glutamyl transferase (carboxy–gamma-glutamyl-p-nitroanilide), alkaline phosphatase (p-nitrophenyl phosphate-diethanolamine), cholesterol (cholesterol oxidase-hydrogen peroxide), high-density lipoprotein cholesterol (phosphotungstic acid), low-density lipoprotein cholesterol (calculated by Friedewald’s formula), triglycerides (glycerol phosphate oxidase-hydrogen peroxide), total protein (Biuret), phosphorus (phosphomolybdic by ultraviolet spectrophotometry) and direct bilirubin (diazosulfanilic acid). For the three remaining tests (total bilirubin, calcium and magnesium), two different methods (A and B) were used by the PLs (Table 1).

**TABLE 1:** Clinical chemistry tests with two different methods used in Togo from July 2016 to December 2016.

Tests	Group A methods		Group B methods	
	Laboratory method	Number of PLs	Laboratory method	Number of PLs
Total Bilirubin	Sulfanilic acid/Dimethylsulfoxyl	10	Sulfanilic acid/Caffeine-sodium benzoate	1
Calcium	O-cresolphthalein	3	Arsenazo III	8
Magnesium	Calmagite	7	Xylidyl Blue	4

PLs, participating laboratories.

Calibration traceability information was stated in the package inserts and all assays were traceable to an appropriate international reference standard or method. Tests were performed by a fully automated or semi-automated spectrophotometer, depending on the analyser available in each PL (Table 2).

**TABLE 2:** Spectrophotometers and brands of analysers and reagents used by the participating laboratories in Togo from July 2016 to December 2016.

Category of spectrophotometer	Manufacturer	Country of origin	Instrument model	Reagents
Fully automated	Vital Scientific^®^	the Netherlands	SELECTRA^®^ PRO M	Elitech^®^
	Spinreact^®^	Spain	SPIN 120^®^	Chemelex^®^ Elitech^®^
	QUIMICA CLINICA^®^	Spain	QUIMICA CLINICA^®^ BIO 100	Quimica clinica^®^
	HUMAN Diagnotics Wordwide^®^	Germany	HUMASTAR 200	Human^®^
	Mindray^®^	China	MINDRAY^®^ BS-120	Chemelex^®^
**Semi-automated**	ROBERT RIELE^®^	Germany	RIELE^®^ 5010	Chemelex^®^
	Hospitex Diagnostics^®^	Italy	Master T- HOSPITEX^®^	Cypress^®^
	URIT^®^	China	URIT^®^-810	Chemelex^®^ Biolabo^®^
	Sinnowa Medical Science & Technology^®^	China	SINNOWA^®^ BS-3000	Chemelex^®^

The sample analysed by each PL consisted of a lyophilised multiparametric serum supplied by OWA. In the four cycles of this pilot programme, the PLs received PT samples of different ranges in each cycle. Five millilitre vials of PHILCO WATER 5^®^ brand distilled water (Philco Pharma Carsten, Grosshansdorf, Germany) was provided for the reconstitution of samples using a volumetric micropipette.

### Proficiency test data collection

Participating laboratories were instructed to test each sample in the same manner as patient samples. Results were documented and sent to OWA for analysis, with the final PT reports typically being received from OWA within 15 days of sample receipt in Togo. A WhatsApp group (WhatsApp LLC, Menlo Park, California, United States) including all 11 PLs and staff from the coordinating team was created to monitor the timely submission of results and PT reports. This WhatsApp group was also used to discuss the implementation of corrective actions when a result was unacceptable or when a PL had issues with continuing the programme. The corrective actions were tracked using a ‘non-conformity management sheet’.

### Data evaluation and performances criteria

The PLs were divided into two main groups to determine and compare their PT performance: one group of PLs used fully automated spectrophotometers and the second group used semi-automated spectrophotometers and volumetric micropipettes. In addition, the performance of labs that used IQC was compared with that of labs that did not, evaluating the impact of IQC. The performance measurements included the overall performance of PLs by cycle, the performance of the PLs in each test across the four cycles, the performance of labs using automated or semi-automated spectrophotometers, and the adherence to the IQC process as identified through the WhatsApp group discussions.

The evaluation criteria were based on the Clinical Laboratory Improvement Amendments (CLIA) acceptable limits. This acceptable limit corresponded to the OWA peer group mean ± (allowable total error) defined by CLIA.^13^ The total error of all the 18 tests was given in plus or minus percentage (± %) except for urea and calcium, which were expressed as an absolute value.^2,14^ The PT reports were qualified as acceptable when the results of the test provided by the PL were within the acceptability limits. For tests where there were no CLIA criteria, such as gamma-glutamyl transferase and direct bilirubin, OWA used the peer group mean ± 2 s.d. (standard deviation) to determine the acceptability limits. Allowable error rates were determined by OWA for each test and stated on the PT reports for each PL during each cycle.

### Statistical data analysis

Results of all PLs were sent both to PLs and directly to the Ministry of Health Division of Laboratories by OWA in a Microsoft Excel file (Microsoft, Redmond, Washington, United States), with the quantitative results of the PLs identified as compliant or non-compliant. The PT results of each PL, with the assigned values of each test, were also checked by the PT coordinating team as recommended in the ISO 13528 guideline.^15^

The data were collated and analysed using Epi-Info software version 3.5.3 (2011, Centers for Disease Control and Prevention, Atlanta, Georgia, United States). The calculation of the number of compliant results for a test or an identified group was used to determine the compliance rates in percentage (%). The performance rates of identified groups were compared using the uncorrected Chi-square test or Fisher’s exact test where appropriate. The same statistical method was used in comparing the number and percentage of acceptable results between two successive cycles.

The target score of acceptable results was 80% as recommended by the World Health Organization Regional Office for Africa.^16^ A cycle participation rate of 100% was expected from all PLs. The *p*-value significance level was < 0.05.

## Results

### Laboratory participation rate

Each PL submitted results after performing all 18 tests. A cycle participation rate of 100% was obtained by nine PLs (82%). The participation rate for the two remaining PLs was 50% for PL4 and 25% for PL9.

### Overall analytical performance of participating laboratories in performing tests

**FIGURE 1:**
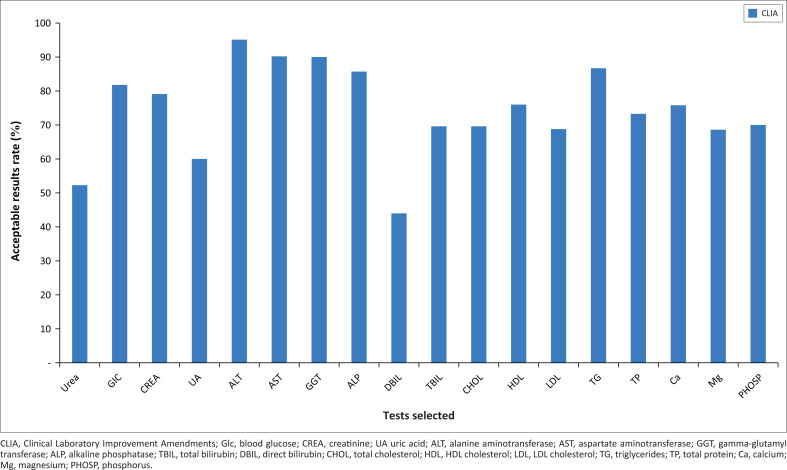
Analytical performance score of participating laboratories for each test performed in Togo from July 2016 to December 2016.

Seventy-six per cent of 775 results were acceptable. The performance scores for urea and direct bilirubin tests were less than 60%. The PLs had a performance score above 80% for blood glucose, alanine aminotransferase, aspartate aminotransferase, gamma-glutamyl transferase, alkaline phosphatase and triglycerides (Figure 1).

### Comparing the overall analytical performance between cycles

Assessing the overall performance level by cycle provides a measurement for the rate of improvement. The performance score increased from 71% to 81% (Table 3). Eight corrective actions were implemented at least once during the four cycles: providing a refrigerator thermometer to monitor the cold chain for better preservation of reagents and samples; planning a daily temperature measurement and performing root cause analysis for any observed deviations relating to out-of-range temperatures; purchasing new refrigerators suitable for laboratory use; calibrating micropipettes; performing periodic preventive maintenance on instruments and corrective maintenance when required; running IQC after each maintenance and re-calibrating instruments when necessary; implementing the use of IQC periodically; and analysing the results of the IQC using a Levey-Jennings chart.

**TABLE 3:** Comparison of participating laboratories’ performance between cycles in Togo from July 2016 to December 2016.

PT cycles	Acceptable results				*p*
	*n*	%	*n*	%	
Cycle 1 versus Cycle 2	156	71	139	78	0.092
Cycle 2 versus Cycle 3	139	78	156	76	0.721
Cycle 3 versus Cycle 4	156	76	141	81	0.287

Note: Cycle 1 (71%) versus Cycle 2 (78%) versus Cycle 3 (76%) versus Cycle 4 (81%); *P* = 0.069.

PT, proficiency test; *n*, number of acceptable results.

### Comparing participating laboratories’ performance based on the use of internal quality control

Out of the 11 PLs, four (36%) used control sera before performing each test, but none of the sites calculated uncertainty. During cycles 2, 3 and 4, the acceptable results rate of PLs using IQC serum was significantly higher than for those not using IQC (Table 4).

**TABLE 4:** Comparison of participating laboratories’ performance based on the use of internal quality control in Togo from July 2016 to December 2016.

PT cycle	Acceptable results				*p*
	PLs using IQC serum (*N* = 4)		PLs using no IQC serum (*N* = 7)		
	*n*	%	*n*	%	
Cycle 1 (*n* = 221)	61	76	95	67	0.110
Cycle 2 (*n* = 177)	55	86	81	71	0.020
Cycle 3 (*n* = 204)	66	89	94	72	0.010
Cycle 4 (*n* = 173)	62	98	87	79	0.002

PT, proficiency test; PL, participating laboratory; *N*, Number of laboratories; *n*, number of results; IQC, internal quality control.

### Rate of acceptable results based on the spectrophotometer category

Five out of 11 PLs used a fully automated spectrophotometer. Three hundred and thirteen (89%) out of 352 results produced were acceptable, against 292 (69%) acceptable results (*p* = 0.001) in the six PLs that used semi-automated spectrophotometers.

## Discussion

This study assessed how well 11 government hospital laboratories performed 18 basic clinical chemistry tests and identified areas needing improvement may be required. This assessment focused on the most common clinical chemistry laboratory tests and therefore a logical starting point for national PT activities.^17^ In Togo, EQA initiatives have only been possible with external funding, and are currently available for tuberculosis, malaria and HIV testing.^18^ On 12 August 2015, the Togo Ministry of Health issued ministerial decree N°115/2015/MSPS/CAB/SG, which formally adopted ISO/IEC 15189:2012 as the quality management system standards to be met by every clinical laboratory in the country. Article 4 of this decree stipulates that: ‘directors of government hospitals and heads of clinical laboratories are required to comply with the standard requirements laid down in ISO 15189’. Consequently, they must start planning and budgeting for PT activities as part of the national funding priorities to ensure a sustainable PT programme. Planning and budgetting is important and should be improved for a sustainable national PT programme.^5^

The low participation rate of PL9 in this study was caused by the breakdown of their spectrophotometer. Three months was insufficient for PL9 to perform the appropriate corrective action and to participate in subsequent cycles. In addition, PL4 suffered reagentstock-outs for six tests, resulting in low participation. The scenario demonstrates the challenge of PLs in low-income countries to implement corrective actions in a timely fashion, even when root causes are identified.^17,19^ This challenge in the present pilot study could in part be mitigated by including at least a three months delay between PT cycles. Insufficient delay between cycles could also explain the lack of significant improvement between cycles. Implementing corrective maintenance and servicing actions for the spectrophotometers and other equipment needs a high level of commitment from the hospital authorities in charge of clinical laboratories, as recommended by ISO/IEC 15189 standards.^7^ This commitment should be manifested through the provision of resources to ensure the availability of reagents and equipment with efficient maintenance services.^19,20^ In addition, a quality management system to monitor overall laboratory quality, including equipment repair, maintenance and calibration, should be implemented as required by the ISO/IEC 15189 regulations.^16,19^ The routine use of IQC by PLs will also enhance laboratory performance, as shown in this study. The use of IQC material at concentrations equal to or close to clinical decision values results in improved test performance and validates reported results.^7^ The laboratories should consider using independent third-party control materials in place of or in addition to reagent or instrument manufacturer IQC materials. The regular review of IQC data to identify acceptable and unacceptable results as well as trends is a good indicator for measuring laboratory performance, and helps to detect performance trends that may indicate problems in the analytical system.^7,20^ The study results showed that PLs using semi-automated spectrophotometers obtained a significantly lower rate of acceptable results than those using fully automated systems. One likely source of this additional error with semi-automated spectrophotometers is the use of micropipettes to measure sera and reagents. These volumetric micropipettes were not calibrated by an ISO/IEC 17025 accredited laboratory and so may not be dispensing accurate volumes. Measurement instruments should be subject to initial reference calibration and regular recalibrations to monitor performance.^21^ Also, PLs using semi-automated spectrophotometers performed poorly because they failed to maintain correct assay temperature or assay incubation period and used test tubes that may not have been washed properly.^22^

The lower performance in urea testing was also documented in a similar study in Ethiopia that used the same OWA PT panel as used in this study. The performance rate for urea testing in 12 Ethiopian laboratories over six cycles was 21% lower than those obtained in the present study.^23^ The suboptimal performance of the PLs for the urea test in this assessment could be attributed to the infrequent urea calibration when using different batches of reagents. Also, the failure to maintain consistent assay temperature might have contributed to the poor urea testing performance.^22^ Generally, proficiency testing evaluation is often done by a specific instrument group or analytical method used. The PLs studied utilised multiple small instruments and multiple reagent kits that are not likely to fit into a specific peer group. This could lead to poorer PT performance for urea and other tests, because the mean method utilised for comparison may not be optimal.^2,24^

The low-performance scores for both total and direct bilirubin can be attributed to multiple factors, such as failure to maintain consistent assay temperature to which the diazo reaction is sensitive, failure to maintain reagent cold chain, and failure to protect calibrators and specimens from exposure to light, as bilirubin is light sensitive.^25^

### Limitations

The major limitations of this study are the use of multiple instruments and reagent kits by PLs. Other limitations of the study include the few numbers of PLs because of limited funds, and the insufficiently spaced PT cycles that did not allow for corrective actions to be implemented before the subsequent PT cycle. In a future study, the impact of the implementing ISO/IEC 15189 requirements on PLs’ performance will be evaluated with the possibility of benchmarking between the central, intermediate and peripheral health levels.

### Conclusion

This study identified areas for improvement for a national PT programme and also demonstrated the value of such work in Togo. It also identified some tests (urea, creatinine, uric acid, bilirubins, cholesterols, otal protein, calcium, magnesium, phosphorus) for which laboratories must improve their performance. It showed that the use of fully automated spectrophotometers is more likely to lead to reliable test results and demonstrated the need for the use of routine appropriate IQC in all laboratories. It emphasised the necessity to plan cycles with sufficient delay for implementing sustainable corrective actions. The national PT programme should be extended to all clinical laboratories in Togo with three cycles per year and should also target all clinical laboratory disciplines.
